# Diversity and natural selection on the thrombospondin-related adhesive protein (TRAP) gene of *Plasmodium knowlesi* in Malaysia

**DOI:** 10.1186/s12936-018-2423-1

**Published:** 2018-07-27

**Authors:** Md Atique Ahmed, Yee Ling Lau, Fu-Shi Quan

**Affiliations:** 1 Department of Medical Zoology, Kyung Hee University School of Medicine, Seoul, 130-705 South Korea; 2 Department of Parasitology, University of Malaya, Kuala Lumpur, Malaysia; 3 Biomedical Science Institute, Kyung Hee University, Seoul, 130-705 South Korea

**Keywords:** *Plasmodium knowlesi*, Natural selection, Thrombospondin-related adhesive protein, Genetic diversity, Sub-clusters, Haplotypes

## Abstract

**Background:**

*Plasmodium knowlesi* a parasite of the macaques is currently the most common cause of human malaria in Malaysia. The thrombospondin-related adhesive protein (TRAP) gene is pre-erythrocytic stage antigen. It is a well-characterized vaccine candidate in *Plasmodium vivax* and *Plasmodium falciparum,* however, no study has been done in the orthologous gene of *P. knowlesi.* This study investigates nucleotide diversity, haplotypes, natural selection and population differentiation of full-length *pktrap* genes in clinical samples from Malaysia.

**Methods:**

Forty full-length *pktrap* sequences from clinical isolates of Malaysia along with the reference H-strain were downloaded from published databases. Genetic diversity, polymorphism, haplotype and natural selection were determined using DnaSP 5.10 software. McDonald–Kreitman test was conducted using *P. vivax* and *Plasmodium coatneyi* as ortholog sequence in DnaSP 5.10 software. Population genetic differentiation index (*F*_*ST*_) of parasite populations was determined using Arlequin v3.5. Phylogenetic relationships between trap ortholog genes were determined using MEGA 5.0 software.

**Results:**

Comparison of 40 full-length *pktrap* sequences along with the H-strain identified 74 SNPs (53 non-synonymous and 21 synonymous substitutions) resulting in 29 haplotypes. Analysis of the full-length gene showed that the nucleotide diversity was lower compared to its nearest ortholog *pvtrap.* Domain-wise analysis indicated that the proline/asparagine rich region had higher nucleotide diversity compared to the von Willebrand factor domain and the thrombospondin-type-1 domain. McDonald–Kreitman test identified that the ratio of the number of nonsynonymous to synonymous polymorphic sites within *P. knowlesi* was significantly higher than that of the number of nonsynonymous to synonymous fixed sites between *P. knowlesi* and *P. vivax*. The von Willebrand factor domain also indicated balancing selection using MK test, however, it did not give significant results when tested with *P. coatneyi* as an outgroup. Phylogenetic analysis of full-length genes identified three distinct sub-clusters of *P. knowlesi*, one originating from Peninsular Malaysia and two originating from Malaysian Borneo. High population differentiation values was observed within samples from Peninsular Malaysia and Malaysian Borneo.

**Conclusions:**

This study is the first to report on the genetic diversity and natural selection of full-length *pktrap*. Low level of genetic diversity was found across the full-length gene of *pktrap*. Balancing selection of the von Willebrand factor domain indicated that TRAP could be a target in inducing immune response against *P. knowlesi* infections. However, higher number of samples would be necessary to further confirm the findings.

**Electronic supplementary material:**

The online version of this article (10.1186/s12936-018-2423-1) contains supplementary material, which is available to authorized users.

## Background

Malaria is a major public health threat in many parts of the globe and is responsible for half a million deaths annually [1]. *Plasmodium knowlesi*, a simian malaria parasite, is the fifth *Plasmodium* species infecting humans and is an emerging malaria in Southeast Asian countries [2–6]. Among all *P. knowlesi* reported countries, the disease epicenter is in Malaysia with increasing number of human infections reported from Peninsular Malaysia and Malaysian Borneo [4, 7, 8], thereby highlighting the requirement of effective measures for control as well as development of effective vaccines. *P. knowlesi* accounts up to 70–78% of malaria cases in Malaysian Borneo cases [8, 9]. The parasite has a 24-h erythrocytic cycle and thus rapid increase in parasite count has been found to be associated with the development of severe malaria in humans some of which are fatal [3, 9–11]. Microsatellite and genome-based studies from Sarawak, Malaysian Borneo have discovered that there are at least three sub-populations of the parasite in clinical samples and two of the populations were associated with the primary monkey hosts; *Macaca fascicularis* and *Macaca nemestrina* in Malaysian Borneo [12–14]. Additionally, mitochondrial and smaller subunit ribosomal rRNA genes of *P. knowlesi* isolates from humans and macaques also identified two distinct sub-population which grouped geographically to Peninsular Malaysia and Malaysian Borneo [15]. Studies on orangutans from Sabah, Malaysian Borneo indicated that at least two sympatric parasite lineages exists and are rapidly speciating [16].

Genetic diversity exhibited by field isolates within potential candidate antigens is a major challenge towards vaccine development and therefore it is crucial to determine the level of diversity, type of natural selection and its significance towards effectiveness of protective immunity. A recent *P. knowlesi* study showed association of polymorphisms within the merozoite invasion genes (normocyte binding protein xa and xb*, nbpxa* and *nbpxb*) with high parasite counts and disease severity in human infections [17]. A number of blood stage antigens like Duffy binding protein (DBP), merozoite surface protein 1 (MSP-1), MSP-1 paralog, MSP-3 and nbpxa have very recently been studied from *P. knowlesi* clinical isolates but none has been shown to be under positive natural selection [18–21].

The *P. falciparum* circumsporozoite (CS) protein is the only candidate for vaccine development that has reached phase III clinical trials [22]. Although CSP-derived immunogens showed immunity against sporozoites, the recombinant subunit vaccine RTS, S has resulted in limited clinical efficacy in field studies of *P. falciparum* due to extensive diversity observed within field isolates [23]. Diversity within *pkcsp* gene from clinical isolates from Malaysia has been high and the epitope binding regions has been under the influence of positive natural selection [24]. The thrombospondin-related adhesive protein (TRAP) is a Type-I transmembrane micronemal protein in the sporozoites. It has been found essential for both guiding motility as well as invasion to hepatocytes and mosquito’s salivary gland [25, 26]. Disruption of the *P. falciparum* TRAP (PfTRAP) gene by gene-knockout impaired sporozoite gliding motility, salivary gland invasion and sporozoite infectivity [27]. The *P. falciparum* TRAP and its homologue species contains a hydrophobic N-terminal peptide, an integrin-like magnesium binding (or von Willebrand factor A) domain, thrombospondin type I repeats, an acidic proline/asparagine-rich region, hydrophobic transmembrane domain and a cytoplasmic tail [28, 29]. Naturally acquired immune responses to PvTRAP have also been reported in a multi-country study [30]. Recently, clinical trial of multi-epitope based TRAP antigen has shown promising safety and immunogenicity and substantial efficacy with high T cell response till 7 days post challenge in *P. falciparum* infections [31]. Studies on polymorphisms and natural selection acting on the TRAP gene using field isolates of *P. falciparum* and *P. vivax* from different geographical locations have indicated that TRAP might be an important vaccine candidate which is under strong diversifying/positive selection [32, 33]. These studies indicated that TRAP molecule is major target of human immune response to pre-erythrocytic stages of the parasite and thus might serve as a good vaccine candidate. Despite its importance, no study has been done to characterize the *P. knowlesi* TRAP (PkTRAP), which is an ortholog gene.

In this study, the domains of PkTRAP protein were characterized based on the amino acid sequence alignment to its ortholog *P. vivax* TRAP (PvTRAP) and *P. falciparum* TRAP (PfTRAP) sequences. Genetic diversity, natural selection, number of haplotypes, haplotype diversity and population differentiation index *F*_*ST*_ using full-length genes as well as at each of the TRAP domains was determined using 37 clinical isolates and three laboratory lines (along with the H-strain) of Malaysia. The information obtained from this study will be helpful to understand the parasite dynamics in Malaysia and for future rational design and formulation of a pre-erythrocytic vaccine against *P. knowlesi*.

## Methods

### PkTRAP sequence data

PkTRAP sequences were downloaded from published database for 37 clinical isolates originating from Malaysian Borneo, 3 long-time isolated lines from Peninsular Malaysia along with the H-strain (PKNH_1265400) (Additional file 1) [14]. Signal peptide for the full-length PkTRAP gene was predicted using Signal IP 3.0 and the trans membrane domains using the Phobious prediction software [34, 35]. All DNA sequences were aligned using the CLUSTAL-W program in MegAlign Lasergene v 7.0 (DNASTAR) and exported in FASTA format for polymorphic and phylogenetic analyses in MEGA 5.0 software. In order to determine the relationship between Pk TRAP from all the geographical location in the study, phylogenetic analyses was conducted using deduced amino acid sequences from 10 PkTRAP full-length from Malaysian Borneo, 1 laboratory lines from Peninsular Malaysia; reference H-strain (PKNH_1265400) and the Malayan Strain (PKNOH_S09533500) (Additional file 1) along with other ortholog members of *P. vivax* Sal-1 (PVX_082735), *Plasmodium cynomolgi* (PcYM12211400), *Plasmodium ovale curtisi* (PoCGH0112027200), *Plasmodium malariae* (PmUG0112028900) and *P. coatneyi* (PCOAH_00042390) using unrooted neighbor-Joining (NJ) method also described in MEGA 5.0. Bootstrap replicates of 1000 were used to test the robustness of the trees. In order to determine the relationships of vWFD domain A of the PkTRAP protein from different geographical areas, separate phylogenetic analysis were conducted using NJ method with *P. o. curtisi* (PoCGH0112027200) as an outgroup and 1000 bootstrap replications.

### Sequence diversity and natural selection

Sequence diversity (π), defined as the average number of nucleotide differences per site between two sequences within the sequences, was determined by DnaSP v5.10 software [36]. Number of polymorphic sites, number of synonymous and non-synonymous substitutions, number of haplotypes (H) and haplotype diversity within the *pktrap* sequences were also determined by DnaSP software. Schematic representation of the nucleotide diversity was conducted using the same software with window length 100 bp and step size 25 bp. Test of natural selection was conducted using two approaches; inter-species and intra-species analysis. To test whether the *pktrap* gene is under the influence of natural selection the robust McDonald and Kreitman (MK) test was performed with both *P. vivax* (PVX_082735) and *P. coatneyi* (PCOAH_00042390) *trap* gene as an out group using DnaSP v5.10 software [37]. The test compares the ratio of the number of non-synonymous (Pn) to synonymous (Ps) polymorphic sites within a species to the numbers of non-synonymous (Dn) and synonymous (Ds) substitutions fixed sites between species per locus. Under neutrality the ratio of Dn/Ds mutations within species should be equal to Pn/Ps between species polymorphisms. However, if the ratio of fixed Dn/Ds between species is less than Pn/Ps within species, the gene is said to be under diversifying selection. Natural selection was also determined at the intra-population level by calculating the rates of synonymous substitutions per synonymous site (dS) and nonsynonymous substitutions per nonsynonymous site (dN) were computed by using Nei and Gojobori’s method [38] with Juke and Cantor correction and their standard errors of these parameters were estimated by the bootstrap method with 1000 pseudo replicates as implemented in the MEGA 5.0 program [30]. Additionally, the Tajima’s D neutrality tests was performed as implemented in DnaSP v5.10 software. Under neutrality, Tajimas D is expected to be 0. Significant positive Tajima’s D values indicate positive/balancing selection, whereas negative values suggest population expansion or negative selection. Graphical representation of Tajimas D value were determined across the full-length gene with window length 100 bp and step size 25 bp using DnaSP v5.10 software. D value was also determined separately for the von Willebrand factor domain with window length 20 and step size 5 with the same program. Additionally, natural selection acting at the von Willebrand factor domain A was also tested by using codon-based site-by-site analysis to detect codon sites under positive selection at the population level by determining the differences between *d*_N_ and *d*_S_ per site tested using five methods; fixed effects likelihood (FEL), internal fixed effects likelihood (IFEL), random effects likelihood (REL), mixed effects model of evolution (MEME) and fast unbiased Bayesian approximation (FUBAR) methods implemented in the HyPhy package [39].

### Population differentiation

The ARLEQUIN software (version 3.5.1.3, University of Berne, Berne, Switzerland) was used to compute pairwise differences (*F*_*ST*_) between populations i.e., Sarikei (n = 4), Betong (n = 12) and Kapit (n = 12) from Malaysian Borneo and Peninsular Malaysia (n = 4) with 10,100 permutations. Since samples were collected from these four distinct regions of Malaysia, they were considered as four distinct populations. *F*_*ST*_ is a comparison of the sum of genetic variability within and between populations on the basis of the differences in allelic frequencies. *F*_*ST*_ values are interpreted as no (0), low (> 0–0.05), moderate (0.05–0.15), and high (0.15–0.25) genetic differentiation.

## Results

### PkTRAP diversity

**Fig. 1:**
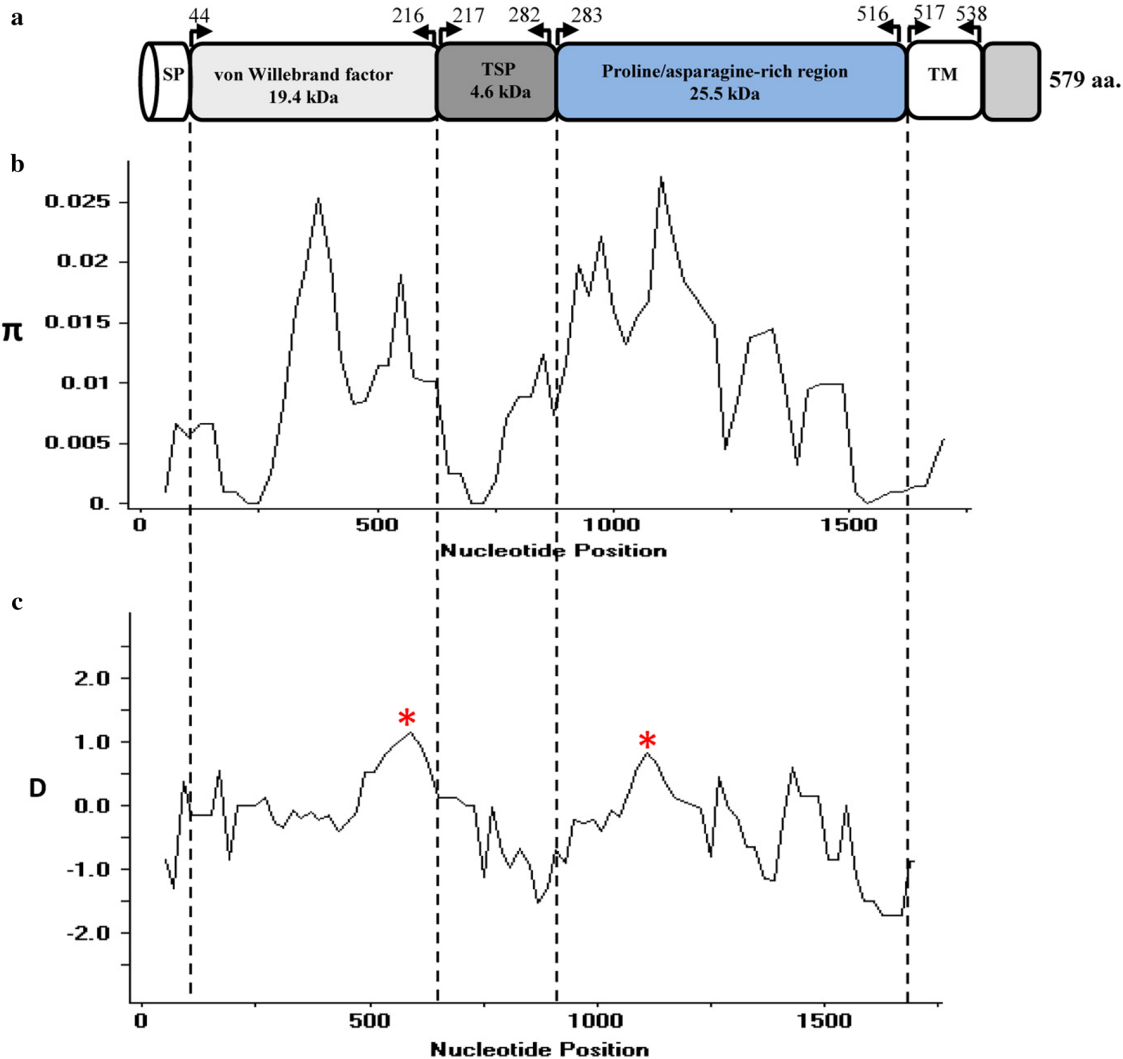
**a** Schematic diagram of *Plasmodium knowlesi* thrombospondin-related adhesive protein (PKNH_1265400) (TRAP). **b** Graphical representation of nucleotide diversity (π) within 40 full-length PkTRAP genes (1740 bp) from Malaysia. The PkTRAP domains are marked above. **c** Graphical representation of Tajima’s D value across the TRAP gene. Asterisk indicates D value which were significant

The signal peptide of the PkTRAP protein was detected between amino acid positions 20 and 30 using the Signal IP and Phobious servers (Additional file 2). Alignment and comparison of the amino acid sequences of the full-length *P. knowlesi* H reference strain TRAP sequences with its ortholog in *P. vivax* Sal-1 reference strain showed 72.3% identity. Other orthologs in *P. falciparum* 3D7 stain and *P. cynomolgi* M-strain it showed 41.2 and 75.4% sequence identity respectively. The schematic structure of *pktrap* gene is shown in Fig. 1a. Within the full-length *pktrap* sequences (1740 bp, n = 40), there were 74 polymorphic sites (4.25%) leading 21 synonymous and 53 nonsynonymous substitutions. There were 57 parsimony informative sites of which two sites were of three variants and 15 singleton variable sites. In addition to nonsynonymous SNPs, the *pktrap* gene had a repeat region with a non-nucleotide repeat unit encoding Proline–Glutamate–Asparagine (P–E–N) region (Additional file 3). The number of repeats varied from three to six within the isolates. Size variations were observed in nine isolates due to deletion within the P–E–N region with gene length varying in between 1722 and 1740 bp. The overall nucleotide diversity was higher (π = 0.00908 ± SD 0.0006) compared to its ortholog in *P. vivax* [33] (Table 1). Within the *pktrap* genes, the nucleotide diversity at the proline/asparagine rich region was the highest (π = 0.0125 ± 0.001) followed by the von Willebrand factor domain (π = 0.00922 ± 0.0001) and the TSP domain (π = 0.0070 ± 0.00095) (Table 1). The sliding window plot (window length 100 bp and step size 25 bp) also revealed that the overall diversity range from 0 to 0.025 and the TSP region containing the showed lower diversity while the von Willebrand factor domain and the proline/asparagine rich region having higher diversities (Fig. 1b). The haplotype numbers as well as the haplotype diversity of the proline/asparagine rich region was high compared to the von Willebrand factor domain and the TSP domain (Table 1). The nucleotide and amino acid polymorphisms observed within the 40 PkTRAP are shown in Additional files 3 and 4 respectively. The 29 *pktrap* haplotypes identified in the study are listed in Additional file 5.

**Table 1:** Estimates of nucleotide diversity, haplotype diversity and neutrality indices of *pktrap*

Domain	No. samples	SNPs	Syn	Non Syn	No. haplotype	Diversity ± SD		Taj D	dN–dS ± S.E	Fu and Li’s D*	Fu and Li’s F*
						Haplotype	Nucleotide				
Full-length	40	74	21	53	29	0.978 ± 0.007	0.00908 ± 0.0007	− 0.38	− 0.003 ± 0.001	0.30	0.07
von Willebrand factor domain		20	4	16	14	0.910 ± 0.023	0.00922 ± 0.0001	0.07	− 0.001 ± 0.001	1.31	1.06
TSP domain		6	1	5	7	0.670 ± 0.054	0.0070 ± 0.00095	− 0.97	− 0.016 ± 0.02	− 0.46	− 0.72
Proline/asparagine-rich region		39	13	26	22	0.959 ± 0.015	0.01254 ± 0.0011	− 0.29	− 0.005 ± 0.001	0.46	0.24

*SNPs* single nucleotide polymorphisms, *SD* standard deviation, *Syn* synonymous substitutions, *NonSyn* non synonymous substitutions, *S.E* standard error

### Natural selection of PkTRAP

**Fig. 2:**
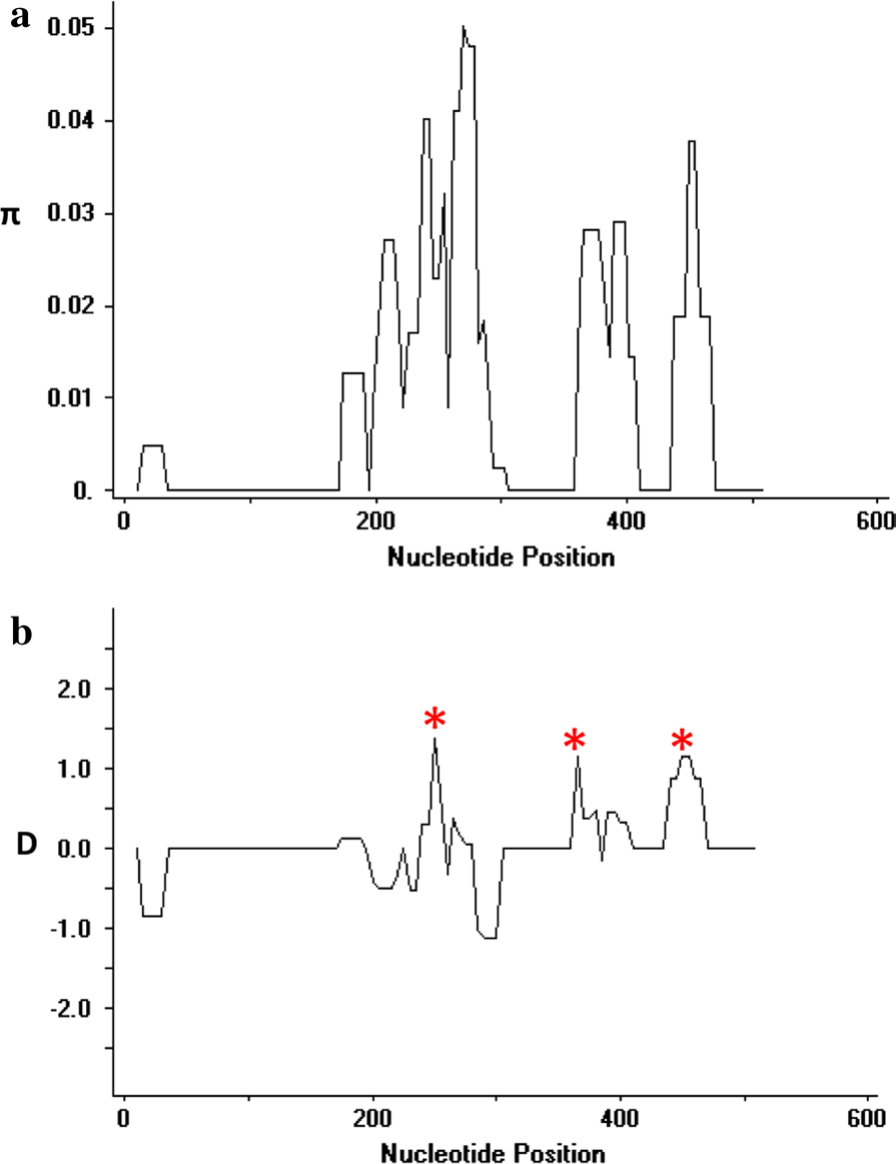
**a** Graphical representation of nucleotide diversity (π) within the von Willebrand factor and **b** Tajimas D value within PkTRAP genes drawn with window length 20 and step size 5 in Dnasp. The asterisk indicates positive Tajimas D values within the domain

Analysis of the full-length genes using the MK test showed that the ratio of the number of nonsynonymous to synonymous polymorphic sites within *P. knowlesi* (53/21) was significantly higher than that of the number of nonsynonymous to synonymous fixed sites between *P. knowlesi* and *P. vivax* (174/144, *P* value = 0.01 by Fisher’s exact test) indicating the *pktrap* is probably under balancing selection (Table 2). Further domain wise analysis using MK test indicated that only the von Willebrand factor domain was significantly under the influence of natural selection (*P* value = 0.02 by Fisher’s exact test) (Table 2). Though the neutrality index of the TSP domain (NI = 5.83) was high, the results were not significant (Table 2). MK test with *P. coatneyi* as an outgroup sequence also showed that the ratio of polymorphic nonsynonymous to synonymous sites were higher than fixed sites between *P. knowlesi* and *P. coatneyi* (126/840 but was not significant (Fisher’s exact test *P* value = 0.06). These results of the MK test indicated a significant excess of non-synonymous polymorphism compared with between-species differences for the *trap* gene when tested with *P. vivax*. However, in order to confirm the MK test results, intra-species analysis were conducted. To investigate if the results from the MK tests were supported by a completely independent approach, based on the statistical distribution of nucleotide frequencies within the population regardless of their coding status, Tajima’s test and Fu and Li’s tests were applied. Similar to the MK tests results only the von Willebrand factor type domain showed positive Tajima’s D value (0.07) and positive Fu and Li’s D* (1.31) and F* values (1.06) respectively indicating positive selection at this domain (Table 1). However, dN–dS test did not give any significant results (Table 1). The graphical representation of Tajima’s D across the *pktrap* genes is shown in Fig. 1c. Positive D value peaks were observed within the von Willebrand factor domain and the proline/asparagine-rich region (Fig. 2b). Codon based site by site analysis of the von-Willebrand factor domain A using five methods; fixed effects likelihood (FEL), internal fixed effects likelihood (IFEL), random effects likelihood (REL), mixed effects model of evolution (MEME) and fast unbiased Bayesian approximation (FUBAR) methods identified five codons (V113T, A/E122V, S134A/G, L137M and Q169E) which were under positive selection (Additional file 6). Out of these five codons, two codons (V113T and S134A/G) were found to be significant by at least two methods (Additional file 6).

**Table 2:** McDonald–Kreitman tests on TRAP of *Plasmodium knowlesi* and its domains with *Plasmodium vivax* and *Plasmodium coatneyi* orthologs as outgroup species

TRAP	Polymorphic changes within *P. knowlesi*		Fixed differences between species				Neutrality index	
	Syn	NonSyn	*Pk* vs *Pv*		*Pk* vs *Pco*		*Pk* vs *Pv*	*Pk* vs *Pco*
			Syn	NonSyn	Syn	NonSyn		
Full-length	21	53	144	174	84	126	2.10*	1.68^$^
von Willebrand factor domain	4	16	47	50	24	38	3.76*	2.52
TSP domain	1	5	14	12	6	3	5.83	1.00
Proline/asparagine region	13	23	47	99	32	74	0.84	0.86

*Syn* Synonymous substitutions, *NonSyn* non synonymous substitutions

* Fisher’s exact test *P* value <* 0.05*

^$^ Fisher’s exact test P value = 0.06

### Phylogenetic analysis

**Fig. 3:**
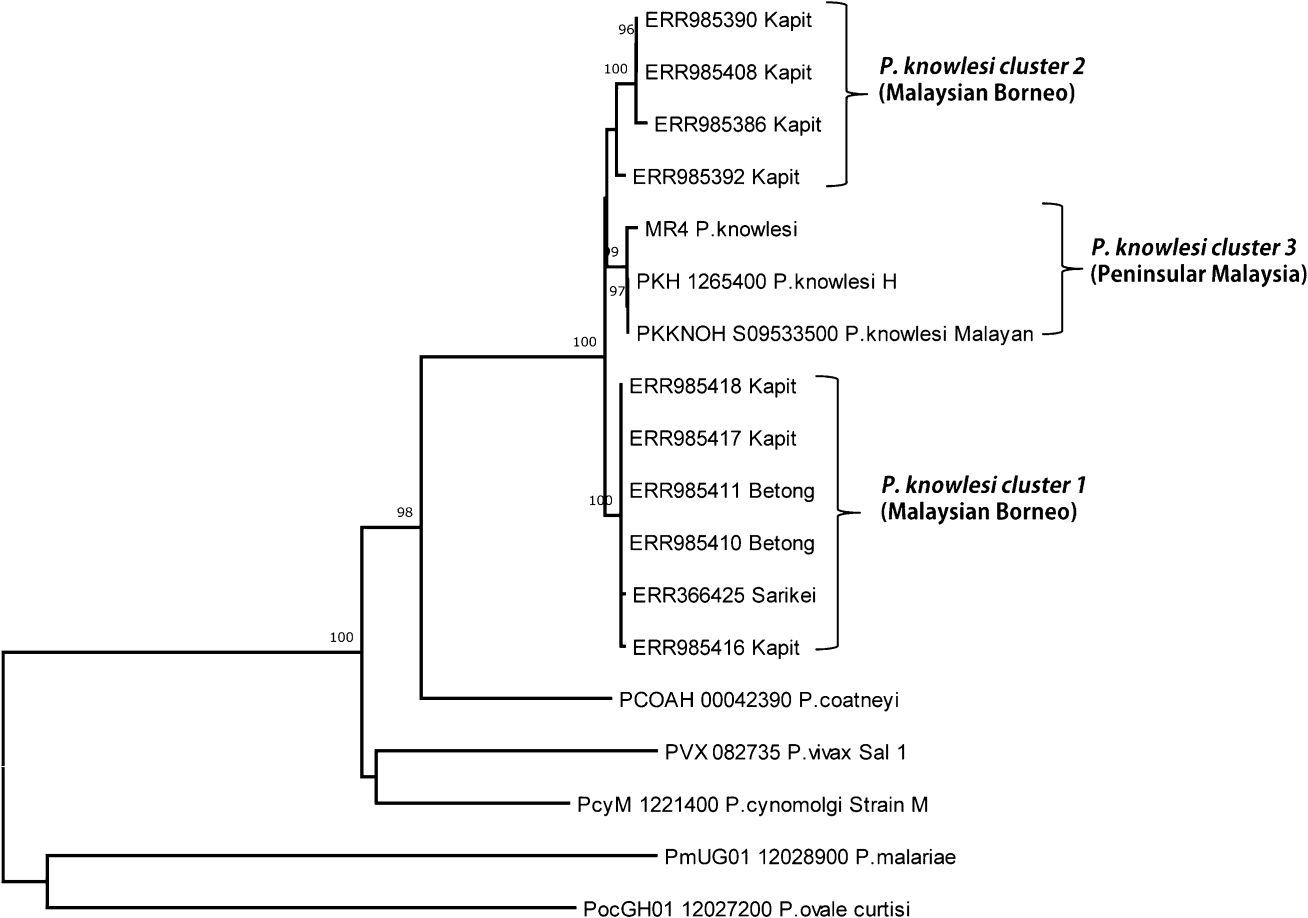
Phylogenetic relationship of TRAP proteins within ortholog *Plasmodium* species *Plasmodium vivax, Plasmodium falciaprum, Plasmodium malariae, Plasmodium knowlesi, Plasmodium ovale* and *P. cynomolgi* based on unrooted neighbor-joining method. The two *P. knowlesi* PkTRAP clusters identified in Malaysian Borneo are shown as cluster 1 and cluster 2 and the two laboratory lines formed the cluster 3 from Peninsular Malaysia. Numbers at the nodes indicate bootstrap values

**Fig. 4:**
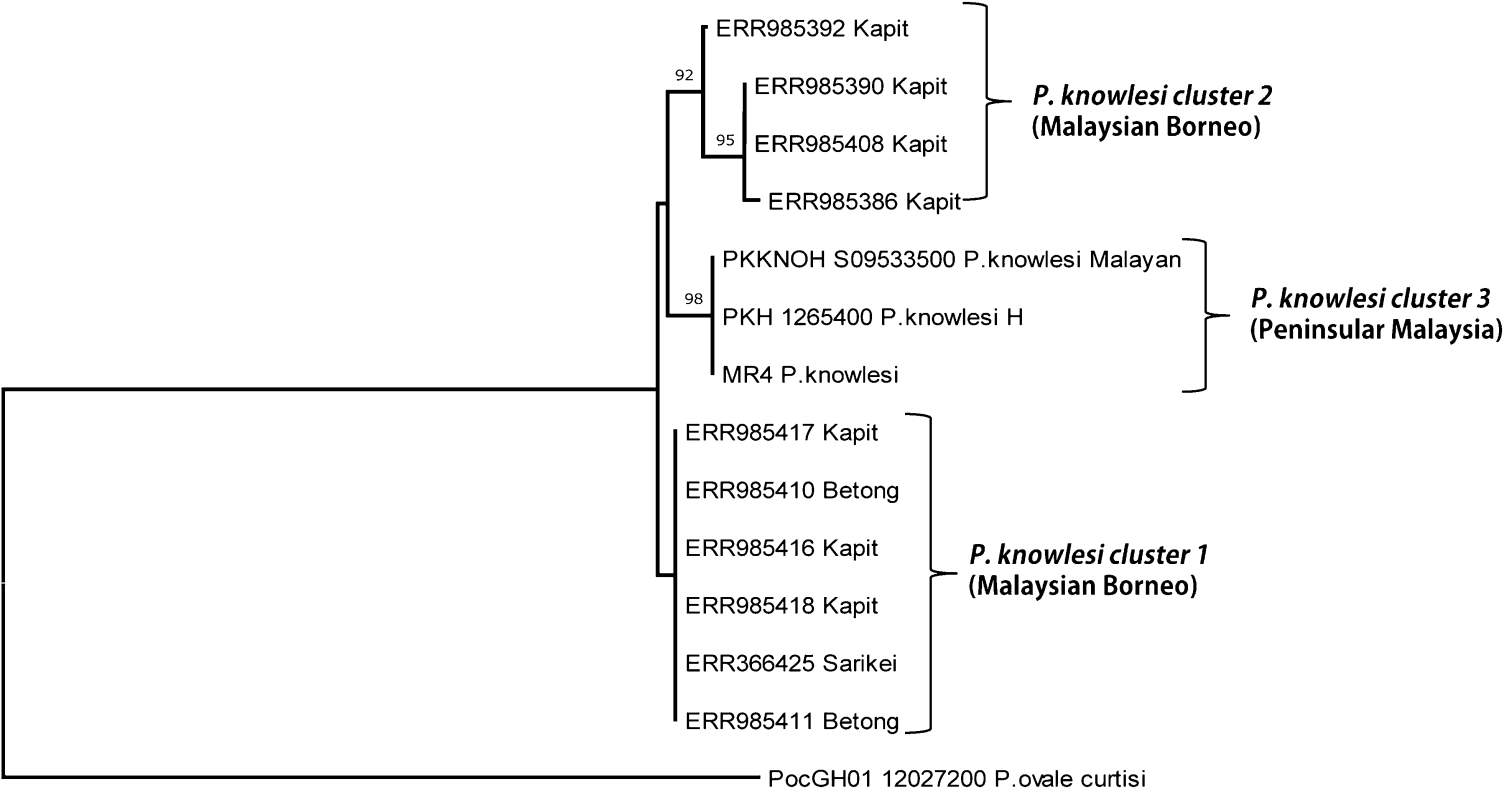
Phylogenetic relationship of von Willebrand factor domain A of the PkTRAP protein from different geographical areas of Malaysia based on neighbour-joining method. The two *P. knowlesi* PkTRAP clusters identified in Malaysian Borneo are shown as cluster 1 and cluster 2 and the two laboratory lines formed the cluster 3 from Peninsular Malaysia. Numbers at the nodes indicate bootstrap values. *Plasmodium ovale curtisi* TRAP was used as an outgroup

Phylogenetic analysis of the 10 full-length PkTRAP deduced amino acid sequences with other *Plasmodium* species using unrooted NJ method identified two distinct *P. knowlesi* clusters from Malaysian Borneo which were supported by 99–100% bootstrap values (Fig. 3a). The two laboratory lines the H-strain and the Malayan Strain, which originated from Peninsular Malaysia formed the third cluster (Fig. 3a). These distinct sub-clusters were similar to the previous discovery of two distinct clusters of sympatric *P. knowlesi* parasites in clinical isolates from Sarawak, Malaysian Borneo at the genomic level [14]. The NJ method showed that the PkTRAP was more closely related to *P. coatneyi* TRAP compared to its ortholog in *P. vivax* and other species (Fig. 3a). NJ tree constructed using the vWFD domain A of sequences from Malaysian Borneo and Peninsular Malaysia also indicated that the dimorphism is intact within this region too (Fig. 4).

### Population differentiation

Pairwise population differentiation index *F*_*ST*_ values using ARLEQUIN software showed high genetic differentiation within the parasite populations originating from Peninsular Malaysia and Malaysian Borneo (Sarikei, Betong and Kapit) and *F*_*ST*_ values were in the range of (0.559–0.676*, P *< *0.000*) (Table 3). This was because of the geographical distance between these locations due to the presence of the South China Sea between Peninsular Malaysia and Malaysian Borneo. However, moderate to very low genetic differentiation was observed between parasites populations within Malaysian Borneo and the *F*_*ST*_ values were in the range of 0.001–0.06*, P *> *0.05* (Table 3). These results indicated that parasitic transmission might be confined to each of the regions i.e., Peninsular Malaysia and Malaysian Borneo but higher number of samples would be required to confirm these test results.

**Table 3:** Population differentiation values (*F*_*ST*_) for *pktrap* from Peninsular Malaysia, Sarikei, Betong and Kapit

Location	*F*_*ST*_ values*			
	Peninsular Malaysia	Sarikei	Betong	Kapit
Peninsular Malaysia	–	–	–	–
Sarikei	0.676*	–	–	–
Betong	0.590**	0.063	–	–
Kapit	0.559**	0.010	0.002	–

** P < 0.001, * P < 0.05

## Discussion

The PfTRAP is one of the major sporozoite antigens that has been reported to generates protective immune response in adults till 7 days post immunization with high T cell response in a clinical trial in Senegal [31]. However, high polymorphisms acting at crucial domains play a decisive role in the efficacy of the vaccine candidate in the field. No study is done on its ortholog. Thus, in the present study the objective was to genetically characterize the *pktrap* gene and study the level of genetic diversity, natural selection acting at the full-length PkTRAP and at its domains from clinical isolates of Malaysia. Sequence alignment of 40 full-length amino acid sequences of *pktrap* genes from Malaysia showed that it shares approximately 72.3% sequence identity with its ortholog *pvtrap*. The nucleotide diversity was higher compared to its nearest ortholog species *P. vivax*. This higher diversity might be probably due to the presence of admixture of *P. knowlesi* sub-populations infecting humans in the Malaysian Borneo [13, 20]. Domain-wise analysis of PkTRAP indicated that the density of the non-synonymous SNPs was higher within the proline/asparagine rich region (SNPs = 22) than the von-Willebrand factor (SNPs = 14) domains. This finding was similar to the findings of PfTRAP from Thailand [32]. However, the ratio of non-synonymous to synonymous SNPs was highest within the von-Willebrand factor domain indicating the region to be under high natural selection pressure. Test of natural selection using both inter and intra-species tests (MK, Taj’s D and Li and Fu’s D* and F*) test indicated that the von-Willebrand factor is probably under balancing selection and might be under the influence of host immune pressure. Similar reports of diversifying selection of PfTRAP and PvTRAP in field isolates has been found in clinical isolates of varied geographical locations [32, 33]. Based on the MK test results the full-length PkTRAP gene also appeared to be under the influence of natural selection however, intra-specific neutrality tests did not yield significant and reliable results (Taj D = − 0.38). Sliding window analysis of Taj D values and diversity across the von-Willebrand factor domain of *pktrap* identified that regions that had higher non-synonymous SNPs also had higher positive values for Taj D indicating that these regions might be possible epitope regions, which are under high selection pressure. A similar study where positive peaks for Taj D values for TRAP has been reported within the CTL epitope regions for *P. falciparum* [40]. Interestingly, MK tests did not show strong significant results when *P. coatneyi* was used as an outgroup sequence (NI = 1.68, P = 0.06) probably because of the presence of dimorphism among the *P. knowlesi* sub-populations. These indicate that higher number of samples would probably result in significant MK test. However, codon based site by site analysis did identify five sites which could be potentially under positive/balancing selection. Since these sites were identified in the region where Tajima’s D value had high peaks, these could potentially be the epitope regions within the von Willebrand factor domain A and to confirm these higher numbers of sequences would be required. Pairwise population differentiation index *F*_*ST*_ values showed high genetic differentiation within the parasite populations originating from Peninsular Malaysia and Malaysian Borneo. These results are similar to previous findings at the genomic level as well as for specific invasion genes [14, 20].

The NJ based phylogenetic tree showed separation of the *P. knowlesi* TRAP genes from Malaysian Borneo into two clusters while the three laboratory lines (H-strain, Malayan strain and the MR4 strain) from Peninsular Malaysia formed a third cluster. Earlier studies on *P. knowlesi* blood stage vaccine candidates such as the DBPαII (PkDBPαII) [41], PkNBPXa [20], PkAMA1 [42] and also a genomic study [14] from Borneo have also reported similar bifurcation of trees. A population genetic study based on microsatellite markers of *P. knowlesi* in humans and macaques indicated that this deep dimorphism was linked to infections from the two natural host the long tailed (*Macaca fascicularis*) and the pig tailed (*Macaca nemestrina*) macaques [13] and humans are susceptible to infections through the both natural hosts. Interestingly, among the *P. knowlesi* pre-erythrocytic vaccine candidates studied to date balancing selection is observed only in TRAP gene thereby highlighting that this molecule might be under effective immune selection and thus could be studied as candidate for vaccine design. Thus, studies are necessary to assess the diversity as well as functional studies directed towards immune response in patient samples would be necessary. However, a cautioned approach is necessary as extensive diversity observed in antigens [43] could be the reason for vaccine failure in the field.

## Conclusions

The present study is the first to investigate genetic diversity and natural selection of the *pktrap* gene from clinical samples. Higher level of genetic diversity was observed in the proline/asparagine rich region of the gene compared to the other domains. The von Willebrand factor domain appeared to be under balancing selection indicating probable host immune pressure in the domain. High genetic differentiation within the parasite populations originating from Peninsular Malaysia and Malaysian Borneo were observed in this study. Future studies should investigate the diversity of TRAP among *P. knowlesi* isolates from all over Malaysia as well as functional studies directed towards development of vaccine strategies.

## Additional files

**Additional file 1: Table S1.** Accession number of PkTRAP sequences used in the study and their geographical origin.

**Additional file 2: Figure S1.** Signal peptide and trans membrane prediction by (A) Signal IP server and (B) Phobious server. Signal peptide was predicted in between amino acid positions 20 to 30.

**Additional file 3: Figure S2.** Amino acid polymorphism within 40 PkTRAP sequences from Malaysia and the P-E-N repeat region.

**Additional file 4.** Nucleotide polymorphism of full-length PkTRAP sequences from Malaysian Borneo.

**Additional file 5.** List of the 29 haplotypes identified within the *pkrap.*

**Additional file 6.** Natural selection analysis of the von Willebrand factor domain A (173 codons) within 41 sequences using the FEL, IFEL and REL, MEME and FUBAR methods.

## Abbreviations

TRAP: thrombospondin-related adhesive protein
nbp: normocyte binding
DBP: duffy binding protein
MSP: merozoite surface protein
CS: circumsporozoite
kDa: kilodalton
bp: base pair
