# Supplementary material for: Diversity and natural selection on the thrombospondin-related adhesive protein (TRAP) gene of Plasmodium knowlesi in Malaysia
Source: Malar J. 2018 Jul 27;17:274. doi: 10.1186/s12936-018-2423-1 (PMC6062916; doi:10.1186/s12936-018-2423-1)
Supplement: Supplementary file 1 — Additional file 1: Table S1. Accession number of PkTRAP sequences used in the study and their geographical origin. [file 12936_2018_2423_MOESM1_ESM.docx]

Additional file 1. Study samples and origin

| No. | Sample | Area | Year |
| --- | --- | --- | --- |
| 1 | ERR274221 | Sarikei | 2012/3 |
| 2 | ERR274222 | Sarikei | 2012/3 |
| 3 | ERR366425 | Sarikei | 2012/3 |
| 4 | ERR366426 | Sarikei | 2012/3 |
| 5 | ERR985374 | Betong | 2012/3 |
| 6 | ERR985376 | Betong | 2012/3 |
| 7 | ERR985377 | Betong | 2012/3 |
| 8 | ERR985378 | Betong | 2012/3 |
| 9 | ERR985379 | Betong | 2012/3 |
| 10 | ERR985380 | Betong | 2012/3 |
| 11 | ERR985381 | Betong | 2012/3 |
| 12 | ERR985382 | Betong | 2012/3 |
| 13 | ERR985383 | Betong | 2012/3 |
| 14 | ERR985384 | Betong | 2012/3 |
| 15 | ERR985385 | Kapit | 2012/3 |
| 16 | ERR985386 | Kapit | 2012/3 |
| 17 | ERR985387 | Kapit | 2012/3 |
| 18 | ERR985388 | Kapit | 2012/3 |
| 19 | ERR985390 | Kapit | 2012/3 |
| 20 | ERR985392 | Kapit | 2012/3 |
| 21 | ERR985393 | Kapit | 2012/3 |
| 22 | ERR985394 | Kapit | 2012/3 |
| 23 | ERR985395 | Kapit | 2012/3 |
| 24 | ERR985396 | Kapit | 2012/3 |
| 25 | ERR985397 | Kapit | 2012/3 |
| 26 | ERR985404 | Kapit | 2012/3 |
| 27 | ERR985405 | Kapit | 2012/3 |
| 28 | ERR985406 | Kapit | 2012/3 |
| 29 | ERR985407 | Kapit | 2012/3 |
| 30 | ERR985408 | Kapit | 2012/3 |
| 31 | ERR985409 | Kapit | 2012/3 |
| 32 | ERR985410 | Betong | 2012/3 |
| 33 | ERR985411 | Betong | 2012/3 |
| 34 | ERR985416 | Kapit | 2012/3 |
| 35 | ERR985417 | Kapit | 2012/3 |
| 36 | ERR985418 | Kapit | 2012/3 |
| 37 | SRR2222335 | P. Malaysia |  |
| 38 | SRR3135172 | P. Malaysia |  |
| 39 | Malayan Strain Pk1A PKNOH_S06430900 | P. Malaysia |  |
| 40 | H-strain(PKNH_0728800) | P. Malaysia |  |
| 41 | ERR985389 | Kapit | 2012/3 |

P: Peninsular

P: Peninsular
