# Supplementary figures and images for: Diversity and natural selection on the thrombospondin-related adhesive protein (TRAP) gene of Plasmodium knowlesi in Malaysia
Source: Malar J. 2018 Jul 27;17:274. doi: 10.1186/s12936-018-2423-1 (PMC6062916; doi:10.1186/s12936-018-2423-1)

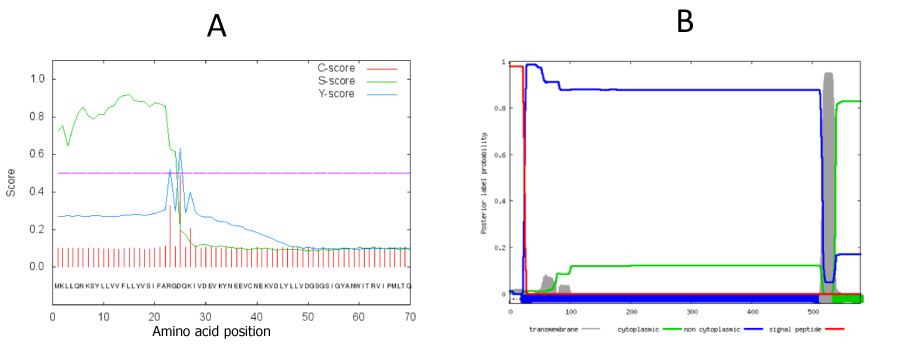

Supplement: Supplementary file 2 — Additional file 2: Figure S1. Signal peptide and trans membrane prediction by (A) Signal IP server and (B) Phobious server. Signal peptide was predicted in between amino acid positions 20 to 30. [file 12936_2018_2423_MOESM2_ESM.jpg]

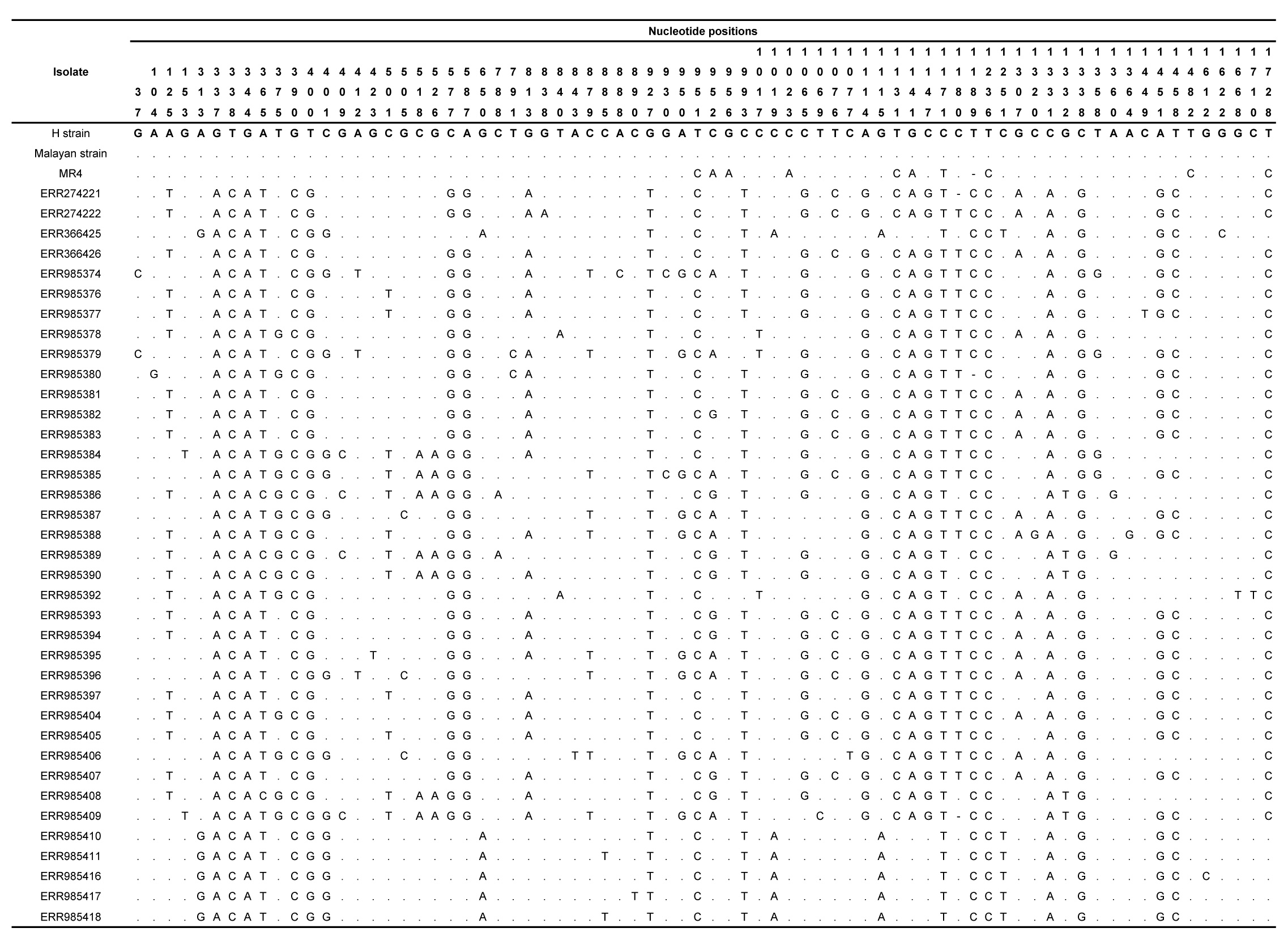

Supplement: Supplementary file 3 — Additional file 3: Figure S2. Amino acid polymorphism within 40 PkTRAP sequences from Malaysia and the P-E-N repeat region. [file 12936_2018_2423_MOESM3_ESM.jpg]

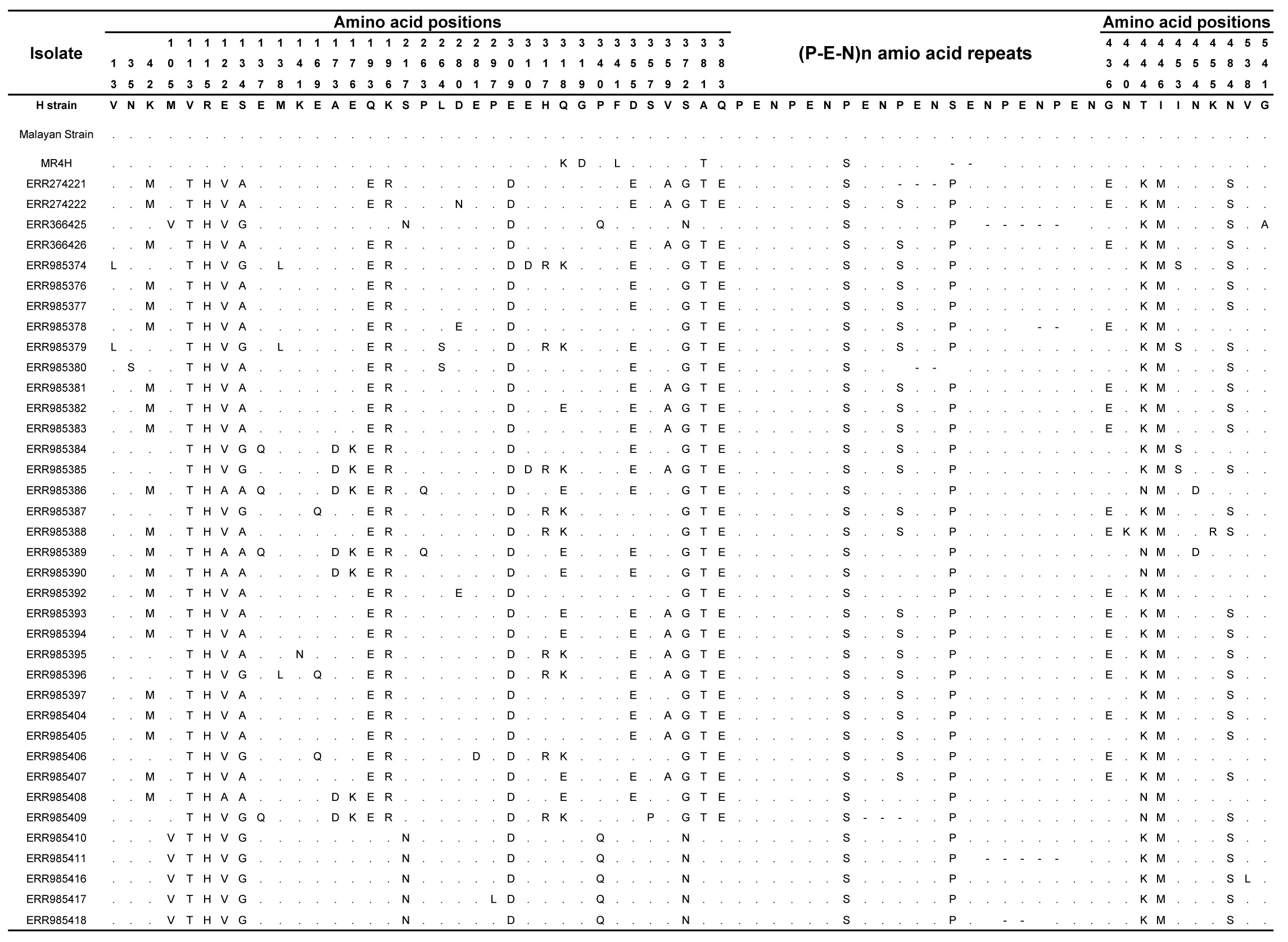

Supplement: Supplementary file 4 — Additional file 4. Nucleotide polymorphism of full-length PkTRAP sequences from Malaysian Borneo. [file 12936_2018_2423_MOESM4_ESM.jpg]
