# Supplementary material for: Diversity and natural selection on the thrombospondin-related adhesive protein (TRAP) gene of Plasmodium knowlesi in Malaysia
Source: Malar J. 2018 Jul 27;17:274. doi: 10.1186/s12936-018-2423-1 (PMC6062916; doi:10.1186/s12936-018-2423-1)
Supplement: Supplementary file 5 — Additional file 5. List of the 29 haplotypes identified within the pkrap. [file 12936_2018_2423_MOESM5_ESM.docx]

| Haplotype No | Haplotype |
| --- | --- |
| Hap_1 | GAAGAGTGATGTCGAGCGCGCAGCTGGTACCACGGATCGCCCCCTTCAGTGCCTCGCCGCTAACATTGGGCT |
| Hap_2 | GAAGAGTGATGTCGAGCGCGCAGCTGGTACCACGGACAACCCACTTCAGCACTCCGCCGCTAACATCGGGCC |
| Hap_3 | GATGAACATTCGCGAGCGCGGGGCTAGTACCACTGACCGTCCCGTCCGGCAGTCCACAGGTAACGCTGGGCC |
| Hap_4 | GATGAACATTCGCGAGCGCGGGGCTAATACCACTGACCGTCCCGTCCGGCAGTCCACAGGTAACGCTGGGCC |
| Hap_5 | GAAGGACATTCGGGAGCGCGCAACTGGTACCACTGACCGTCACCTTCAATGCTCTGCAGGTAACGCTGCGCT |
| Hap_6 | CAAGAACATTCGGGTGCGCGGGGCTAGTATCCCTCGCAGTCCCGTTCGGCAGTCCGCAGGGAACGCTGGGCC |
| Hap_7 | GATGAACATTCGCGAGTGCGGGGCTAGTACCACTGACCGTCCCGTTCGGCAGTCCGCAGGTAACGCTGGGCC |
| Hap_8 | GATGAACATTCGCGAGTGCGGGGCTAGTACCACTGACCGTCCCGTTCGGCAGTCCGCAGGTAATGCTGGGCC |
| Hap_9 | GATGAACATGCGCGAGCGCGGGGCTGGAACCACTGACCGCTCCCTTCGGCAGTCCACAGGTAACATTGGGCC |
| Hap_10 | CAAGAACATTCGGGTGCGCGGGGCCAGTATCACTGGCAGCTCCGTTCGGCAGTCCGCAGGGAACGCTGGGCC |
| Hap_11 | GGAGAACATGCGCGAGCGCGGGGCCAGTACCACTGACCGTCCCGTTCGGCAGTCCGCAGGTAACGCTGGGCC |
| Hap_12 | GATGAACATTCGCGAGCGCGGGGCTAGTACCACTGACGGTCCCGTCCGGCAGTCCACAGGTAACGCTGGGCC |
| Hap_13 | GAATAACATGCGGCAGTGAAGGGCTAGTACCACTGACCGTCCCGTTCGGCAGTCCGCAGGGAACATTGGGCC |
| Hap_14 | GAAGAACATGCGGGAGTGAAGGGCTGGTATCACTCGCAGTCCCGTCCGGCAGTCCGCAGGGAACGCTGGGCC |
| Hap_15 | GATGAACACGCGCCAGTGAAGGGATGGTACCACTGACGGTCCCGTTCGGCAGTCCGCATGTGACATTGGGCC |
| Hap_16 | GAAGAACATGCGGGAGCCCGGGGCTGGTATCACTGGCAGTCCCCTTCGGCAGTCCACAGGTAACGCTGGGCC |
| Hap_17 | GATGAACATGCGCGAGTGCGGGGCTAGTATCACTGGCAGTCCCCTTCGGCAGTCCAGAGGTAGCGCTGGGCC |
| Hap_18 | GATGAACACGCGCGAGTGAAGGGCTAGTACCACTGACGGTCCCGTTCGGCAGTCCGCATGTAACATTGGGCC |
| Hap_19 | GATGAACATGCGCGAGCGCGGGGCTGGAACCACTGACCGCTCCCTTCGGCAGTCCACAGGTAACATTGGTTC |
| Hap_20 | GAAGAACATTCGCGATCGCGGGGCTAGTATCACTGGCAGTCCCGTCCGGCAGTCCACAGGTAACGCTGGGCC |
| Hap_21 | GAAGAACATTCGGGTGCCCGGGGCTGGTATCACTGGCAGTCCCGTCCGGCAGTCCACAGGTAACGCTGGGCC |
| Hap_22 | GATGAACATGCGCGAGCGCGGGGCTAGTACCACTGACCGTCCCGTCCGGCAGTCCACAGGTAACGCTGGGCC |
| Hap_23 | GATGAACATTCGCGAGTGCGGGGCTAGTACCACTGACCGTCCCGTCCGGCAGTCCGCAGGTAACGCTGGGCC |
| Hap_24 | GAAGAACATGCGGGAGCCCGGGGCTGGTTTCACTGGCAGTCCCCTTTGGCAGTCCACAGGTAACATTGGGCC |
| Hap_25 | GAATAACATGCGGCAGTGAAGGGCTAGTATCACTGGCAGTCCCCCTCGGCAGTCCGCATGTAACGCTGGGCC |
| Hap_26 | GAAGGACATTCGGGAGCGCGCAACTGGTACCACTGACCGTCACCTTCAATGCTCTGCAGGTAACGCTGGGCT |
| Hap_27 | GAAGGACATTCGGGAGCGCGCAACTGGTACTACTGACCGTCACCTTCAATGCTCTGCAGGTAACGCTGGGCT |
| Hap_28 | GAAGGACATTCGGGAGCGCGCAACTGGTACCACTGACCGTCACCTTCAATGCTCTGCAGGTAACGCTCGGCT |
| Hap_29 | GAAGGACATTCGGGAGCGCGCAACTGGTACCATTGACCGTCACCTTCAATGCTCTGCAGGTAACGCTGGGCT |

Additional file 5: Twenty nine *pktrap* haplotypes identified in the study
