# Supplementary material for: Diversity and natural selection on the thrombospondin-related adhesive protein (TRAP) gene of Plasmodium knowlesi in Malaysia
Source: Malar J. 2018 Jul 27;17:274. doi: 10.1186/s12936-018-2423-1 (PMC6062916; doi:10.1186/s12936-018-2423-1)
Supplement: Supplementary file 6 — Additional file 6. Natural selection analysis of the von Willebrand factor domain A (173 codons) within 41 sequences using the FEL, IFEL and REL, MEME and FUBAR methods. [file 12936_2018_2423_MOESM6_ESM.docx]

| **Additional file 6. Selection pressure analysis of the 41 *pktrap* von Willebrand factor domain A (173 codons) using the FEL, IFEL and REL, MEME and FUBAR methods** | | | | | | | | | |
| --- | --- | --- | --- | --- | --- | --- | --- | --- | --- |
| **Codon** | **FEL** | | **IFEL** | | **REL** | | **MEME** | **FUBAR** | |
|  | *p* value | *dN-dS* | *p* value | *dN-dS* | Bayes Factor | E[*dN-dS*] | *p* value | *dN-dS* | Posterior Probability |
| **V113T** | 0.189 | 411.18 | - | - | **443.66** | **13.26** | 0.136 | **3.550** | **0.999** |
| A/E122V | 0.213 | 396.8 | - | - | 414.06 | 13.22 | 0.234 | 2.910 | 0.893 |
| **S134A/G** | **0.042** | **946.86** | **0.04** | **1109.1** | **9595.5** | **13.74** | **0.042** | **5.584** | **0.952** |
| L137M | 0.550 | 312.90 | - | **-** | **260.50** | **12.79** | 0.457 | 3.434 | 0.880 |
| Q169E | 0.555 | 313.05 | - | - | **260.61** | **12.79** | 0.452 | 3.430 | 0.889 |

Codons which were found significant at least by two method is in bold
